# Monkeypox Virus Detection in Different Clinical Specimen Types

**DOI:** 10.3201/eid2812.221160

**Published:** 2022-12

**Authors:** Maan Hasso, Stephen Perusini, Alireza Eshaghi, Elaine Tang, Romy Olsha, Hanyue Zhang, Evelyn Lau, Ashleigh Sullivan, Kirby Cronin, Seyeon Lee, Janet Obando, Cedric DeLima, Sandeep Nagra, Tom Braukmann, Venkata R. Duvvuri, Melissa Richard-Greenblatt, Antoine Corbeil, Julianne V. Kus, Anna Majury, Samir Patel, Jonathan B. Gubbay

**Affiliations:** Public Health Ontario, Toronto, Ontario, Canada (M. Hasso, S. Perusini, A. Eshaghi, E. Tang, R. Olsha, H. Zhang, E. Lau, A. Sullivan, K. Cronin, S. Lee, J. Obando, C. DeLima, S. Nagra, T. Braukmann, V.R. Duvvuri, M. Richard-Greenblatt, A. Corbeil, J.V Kus, A. Majury, S. Patel, J.B. Gubbay);; University of Toronto, Toronto (V.R. Duvvuri, M. Richard-Greenblatt, A. Corbeil, J.V. Kus, S. Patel, J.B. Gubbay);; University of Oxford, Oxford, UK (M. Richard-Greenblatt);; Queen's University, Kingston, Ontario, Canada (A. Majury);; The Hospital for Sick Children, Toronto (J.B. Gubbay)

**Keywords:** monkeypox, monkeypox virus, MPXV, viruses, clades, clinical diagnostics, clinical specimen types, preferred specimen source, zoonoses, Canada

## Abstract

A global monkeypox outbreak began in May 2022. Limited data exist on specimen type performance in associated molecular diagnostics. Consequently, a diverse range of specimen sources were collected in the initial weeks of the outbreak in Ontario, Canada. Our clinical evaluation identified skin lesions as the optimal diagnostic specimen source.

The rapid emergence of monkeypox in nonendemic regions of the world during 2022 has health systems on alert (*1*). Monkeypox is a zoonosis caused by monkeypox virus (MPXV) (genus *Orthopoxvirus* [OPXV]). MPXV forms 2 distinct clades: clade I (formerly the Congo Basin/Central African clade), associated with higher virulence and greater mortality rate, and clade II (formerly the West African clade), which is responsible for the current global outbreak (*2*,*3*).

The rapid increase in monkeypox cases in nonendemic areas has challenged clinical laboratories. MPXV shedding and transmission are poorly understood, and relevant data to support clinical management and public health response are lacking. Human-to-human transmission occurs by respiratory droplets, direct contact with skin lesions of infected persons, or contact with contaminated fomites (*4*). Skin lesions, when present, are presumed to be the primary source of viral shedding. Thus, testing of lesion swab specimens by real-time reverse transcription PCR (RT-PCR) is believed to be optimal for diagnosis (*5*,*6*). MPXV can also be detected in other sites, such as throat, nasopharynx, blood, urine, saliva, and semen (*7*).

We investigated detection of MPXV among different clinical specimen types. These specimens were submitted to the provincial reference laboratory for testing in the early weeks of the 2022 outbreak in Ontario, Canada. 

## The Study

The Public Health Ontario Ethics Review Board determined that this study did not require research ethics committee approval because it describes analyses that were completed at the Public Health Ontario laboratory as part of routine clinical testing and surveillance during the monkeypox outbreak in Ontario. Therefore, this study was considered to be public health practice and was exempt from this requirement.

This retrospective study was conducted on patient specimens submitted to Public Health Ontario’s laboratory, the reference microbiology laboratory in Ontario and MPXV testing location, during the initial weeks of the provincial surge (May‒June 2022). All specimens were collected from symptomatic patients with suspected monkeypox infection. Specimen types were categorized as blood, skin lesions, nasal or nasopharyngeal (NP) swab specimens, and oropharyngeal (throat) swab specimens. Urine, semen, and saliva were infrequently submitted. We extracted clinical data submitted on the laboratory requisition, including demographic variables, clinical information, and enterovirus laboratory test results if available.

We extracted DNA from clinical specimens using either the automated total nucleic acid method (NucliSENS easyMAG; bioMérieux, https://www.biomerieux.com) or the manual method (QIAamp DNA Minikit; QIAGEN, https://www.qiagen.com). We conducted testing by using 1 of 2 assays developed by the US Centers for Disease Control and Prevention (CDC): the Laboratory Response Network pan-Orthopoxvirus (OPX) RT-PCR and an MPXV-specific RT-PCR endorsed by the World Health Organization (*8*–*10*). Both assays were validated in-house for clinical testing. The OPXV assay primer/probe set was supplied by CDC for restricted use through the Canadian Laboratory Response Network. The MPXV RT-PCR consists of a generic MPXV GR2-G target (MPX), an MPXV clade II–specific target, and an RNaseP extraction control.

Using the QuantStudio5 RT-PCR system and TaqPath ProAmp Multiplex master mix (Thermo Fisher Scientific, https://www.thermofisher.com), we amplified a 10-μL reaction containing 4 μL DNA, 0.5 μmol/L primers, and 0.2 μmol/L probe. Thermocycling conditions were 60°C for 30 s, 95°C for 5 min, and 45 cycles at 95°C for 5 s and 60°C for 30 s. Cycle threshold (Ct) values <38 was reported as detected, 38.01‒39.99 as indeterminate, and >40 as not detected for MPXV DNA (*11*).

The testing algorithm shifted from the OPXV RT-PCR to the MPXV RT-PCR once the MPXV assay was validated. Some specimens were tested by both assays during validation. RT-PCR results, including Ct values, were evaluated by specimen type among patients who had multiple specimens collected during the same testing episode. Parallel testing for enterovirus by RT-PCR was conducted for pediatric (age <18 years) patients as the most likely differential diagnosis in this age group and for adults (age >18 years) upon request.

We tested 1,063 specimens from 372 patients (mean age 33.8 years, range >1–88 years); 71.2% were male. MPXV was detected in 81 (21.8%) patients, all adult males who had a mean age of 38 years (range 19–65 years). Specimen positivity rate was 23.4% (249/1,063); 2.8% (29/1,063) of all specimens tested had indeterminate results.

Among specimens submitted from the 81 MPXV-positive patients, skin lesions displayed the highest positivity rate (177/213, 83.1%), followed by oropharyngeal (31/46, 67.4%), nasal or NP (20/36, 55.6%), blood (29/67, 43.3%) and urine (6/21, 28.6%) (Table). MPXV was also detected in 2/5 semen specimens and 1/1 saliva specimen submitted from known MPXV-positive patients.

**Table:** Detection results for MPXV by real-time reverse transcription PCR in clinical specimen types submitted to the Public Health Ontario Laboratory, Toronto, Ontario, Canada*

Specimen types and number	Blood, n = 190	Nasal/NP, n = 137	Throat/OP, n = 106	Skin lesions, n = 559	Urine, n = 41
Positive	29 (15.3)	20 (14.6)	31 (29.2)	177 (31.7)	6/41 (14.6)
Specimens from positive patients	29/67 (43.3)	20/36 (55.6)	31/46 (67.4)	177/213 (83.1)	6/21 (28.6)
Target					
Orthopoxvirus					
No. positive	26	16	24	135	5
Mean Ct (SD), range	38.6 (4.1), 27.2‒40.0	36.3 (6.3), 23.3‒38.8	32.0 (6.5), 17.5‒38.0	27.1 (7.3), 14.3‒ 39.6	37.3 (5.1), 29.0‒37.9
Monkeypox					
No. positive	15	11	13	74	4
Mean Ct (SD), range	35.9 (2.1), 32.2‒37.9	32.4 (5.6), 18.2‒37.7	27.8 (5.1), 19.2‒36.0	23.1 (6.5), 12.0‒37.9	32.2 (5.5), 27.2‒37.7
Clade II					
No. positive	15	13	13	75	4
Mean Ct (SD), range	35.3 (2.3), 31.2‒37.1	32.8 (5), 18.1‒37.6	27.3 (4.5), 19.6‒35.2	23.1 (6.6), 11.1‒37.4	32.2 (5.1), 27.7‒ 37.2

*Values are no. (%) except as indicated. Among 30 additional specimens not shown, MPXV was detected in 2/5 semen, 1/4 saliva, 0/1 cerebrospinal fluid, and none of 20 specimens with undocumented sources. Indeterminate results are not included; those are blood 6/190 (3.2%), nasal/NP 5/137 (3.6%), throat/OP 2/106 (1.9%), skin lesion 10/559 (1.8%), and urine 6/41 (14.6%). Ct, cycle threshold; MPXV, monkeypox virus; NP, nasopharyngeal; OP, oropharyngeal.

Across all positive specimens, the MPXV GR2-G target mean Ct (26.2) was 2.7 lower than that of the OPXV assay (29.9), and the clade II target mean Ct (26.3) was 2.6 times lower. The MPXV assay also had lower Ct values than the OPXV assay across all specimen types (Table), indicating higher analytical sensitivity. Skin lesion specimens were detected multiple cycles earlier, indicative of higher viral loads. Oropharyngeal samples had the second lowest Ct means.

Among 78 monkeypox confirmed case-patients with skin and NP or throat swab specimens submitted for testing, 72/78 (92.3%) had >1 positive skin specimen and 38/78 (48.7%) had >1 positive NP or throat swab specimens. MPXV was only detected in skin specimens in 34/78 (43.6%) patients. All patients with a positive blood specimen had >1 other positive specimen type. Among 15 monkeypox confirmed case-patients who had both NP and throat swab specimens submitted, 8 had concordant (53.3%) positive and 2 (13.3%) had concordant negative results. One case-patient had a negative NP swab and positive throat swab specimens, and 4 discordant case-patients had 1 sample type indeterminate and the other negative.

Enterovirus was detected in 25 (71.4%) of the 35 children tested. It was also detected in 7 (46.7%) of the 15 adults tested.

## Conclusions

In this study, MPXV was detected in specimens from multiple sites. Skin lesions most often tested positive, as observed in 92.3% of laboratory confirmed case-patients who had >1 skin specimens tested. This finding indicates that this is the most clinically relevant specimen when skin lesions are available. Positivity rates for other specimen types suggest their use as alternatives if skin lesions are not available. However, the high clinical sensitivity (83.1%) of single skin specimens for MPXV detection suggests that 2 (or 3) appropriately collected specimens from open skin lesions should be adequate for testing. Including additional specimen types probably provides limited value in patients who have lesions. The CDC recommendation of collecting 2 swab specimens from each skin lesion might not be required when >1 skin lesion can be swabbed, which will assist with use of laboratory resources should testing demands increase (*10*). 

Because of our findings, Public Health Ontario advises that specimens other than skin lesions are not required if patients have multiple skin lesions that can be swabbed. However, blood should always be submitted along with NP or throat swab specimens for patients without open skin lesions (e.g., fever without rash or only macular/papular rash). Children are an exception to this strategy because collecting alternative specimens (such as NP swab specimens) enables investigation of other likely etiologies of rash and fever (e.g., respiratory viruses including adenovirus, enterovirus, and rhinovirus).

According to epidemiologic summaries for Ontario, the most common symptoms reported among monkeypox-confirmed case-patients (all tested at the Public Health Ontario laboratory) were rash, fever, lymphadenopathy, oral/genital lesions, and fatigue (*12*). Limitations of this study include lack of detailed clinical information (e.g., symptom onset date) for many patients when not described on the test requisition. Therefore, test performance could not be correlated with disease progression. Further evaluations of different specimen types with detailed temporal data is warranted.
